# Supplementary material for: Molecularly Imprinted Ligand-Free Nanogels for Recognizing Bee Venom-Originated Phospholipase A2 Enzyme
Source: Polymers (Basel). 2022 Oct 7;14(19):4200. doi: 10.3390/polym14194200 (PMC9571764; doi:10.3390/polym14194200)
Supplement: Supplementary file 1 [file polymers-14-04200-s001.zip › polymers-1951226-supplementary.pdf]

Molecularly Imprinted Ligand - Free Nanogels for recognizing bee venom - originated phospholipase A2 enzyme

Anamaria Zaharia#, Ana-Mihaela Gavrila#, Iuliana Caras, Bogdan Trica, Anita-Laura Chiriac, Catalina Ioana Gifu, Iulia Elena Neblea, Elena-Bianca Stoica, Sorin Viorel Dolana, Tanta-Verona Iordache*

Synthesis of difunctional macromer polyethyleneglycol diacrylate (PEGDA, MW=2000 g/mol) according to Radu *et.*al [A. L. Radu, A. M. Gavrila, B. Cursaru, C. P. Spatarelu, T. Sandu, A. Sarbu, M. Teodorescu, F. X. Perrin, T. V. Iordache, **A. Zaharia**; "Poly (ethylene Glycol) Diacrylate-Nanogels Synthesized by Mini-emulsion Polymerization"; **(2019)**, 56, No. 3, **2019**, 514-519].

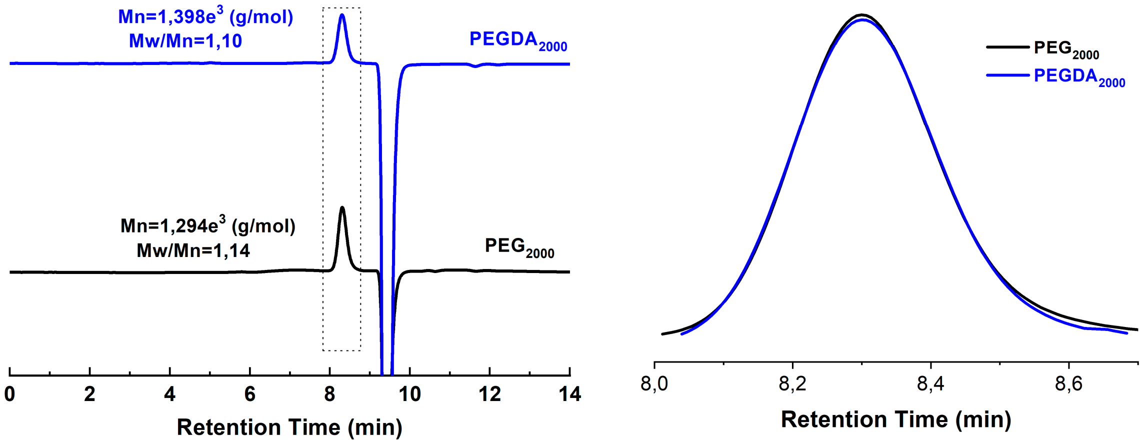

Figure S1: Molecular weight distribution by size exclusion chromatography corresponding to the precursor PEG_{2000} and the synthesized $PEGDA_{2000}$ macromer (the latter showing a slightly higher molecular weight due to acrylate insertion as end groups to the PEG_{2000} precursor)

Further on, by comparing the areas of the peaks corresponding to the protons from the CH2 group (non-equivalent AB system), (δ = 6.42 and δ = 5.83 ppm) with the protons of the oxyethylene units (CH2CH2O) (δ = 3.78 ppm) from the ^{1}H-NMR spectra (**Fig. S2**) the functionality of the synthesized $PEGDA_{2000}$ was determined, using the following equation:

$$\bar{f}(acrylicgroups) = \frac{A_{methylene}}{A_{oxyethylene/4}} \times \overline{DP_{nPEG}}$$

The synthesized macromer presented a functionalization yield of 84 % and a diacrylate functionality of 1.67 PEG. These parameters investigated by the NMR technique were particularly important in the synthesis stage of molecularly imprinted nanogels by inverse emulsion. A macromer with functionality higher than 1.5 will favour the obtaining of more

crosslinked networks that will ultimately increase the thermal and mechanical stability of nanogels and protect the imprinted cavities against physical deformations. Damaging the binding sites may lead to partial recognition of the template and, thus, low specificity.

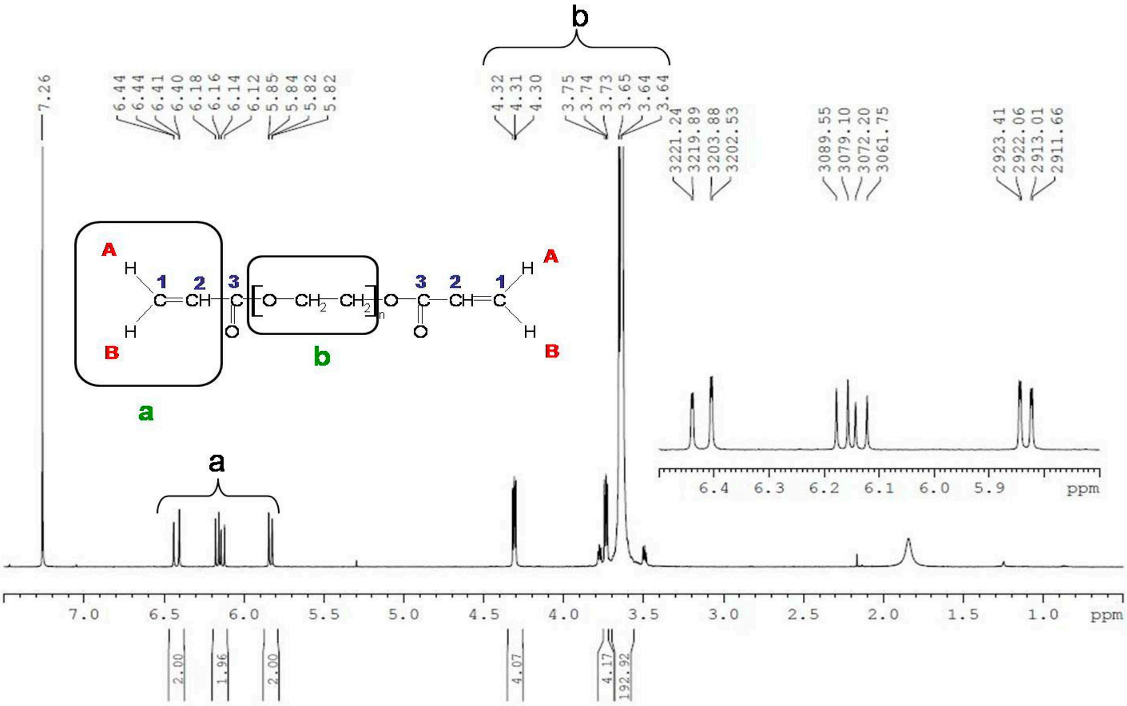

Figure S2: H^1- NMR spectrum of synthesized $PEGDA_{2000}$ macromer

The characteristic FTIR spectrum of the macromonomer obtained by the route of polyethylene glycol and acryloyl chloride ($PEGDA_{2000}$) is shown in **Fig. S3**. Thus, the spectrum presented a broad absorption band at 3464 cm^{-1}, characteristic of the O-H groups in PEG. The band at 2885 cm^{-1} was attributed to the characteristic stretching vibrations of C-H group, while the bands at 1464 cm^{-1} and 1346 cm^{-1} were assigned to the formation vibrations characteristic of the C-H groups. The bands at 1281 cm^{-1} and 1109 cm^{-1} are characteristic for the stretching vibrations corresponding to the O-H and C-O-H groups, respectively, in PEG. Further on, the band at 1725 cm^{-1} was attributed to the protonated carboxylate groups (–C=O), while the band at 1636 cm^{-1} was associated with the asymmetric and symmetric stretching vibrations of - C-O in the same carboxylate groups.

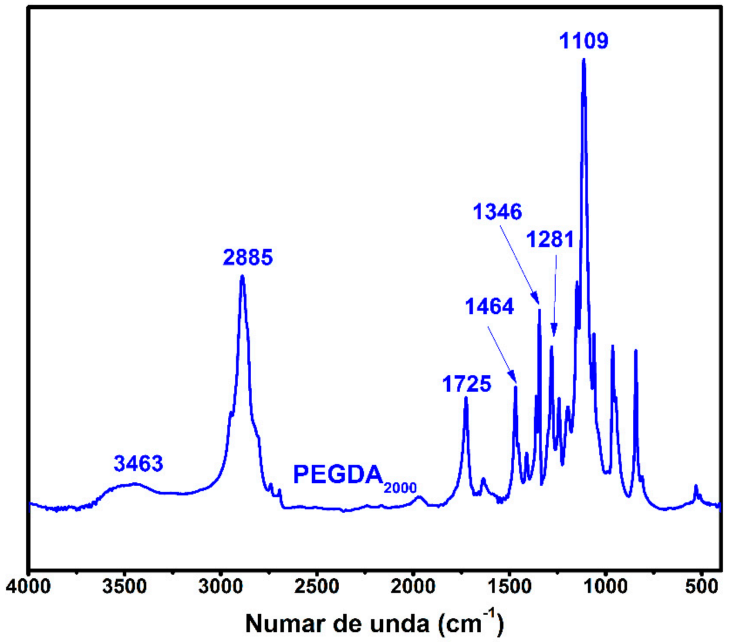

Figure S3. FTIR spectra of synthesized $PEGDA_{2000}$ monomer

Fig. S4 shows the characteristic TGA and DTG curves of the synthesized $PEGDA_{2000}$ macromer. Its thermal behaviour shows a single stage of thermal degradation. Thus, the thermogravimetric analysis recorded a weight loss of 99.99% in the range 303-456 °C, with a maximum decomposition temperature of 395.82 °C. This degradation step was associated with the decomposition process of the PEGDA backbone.

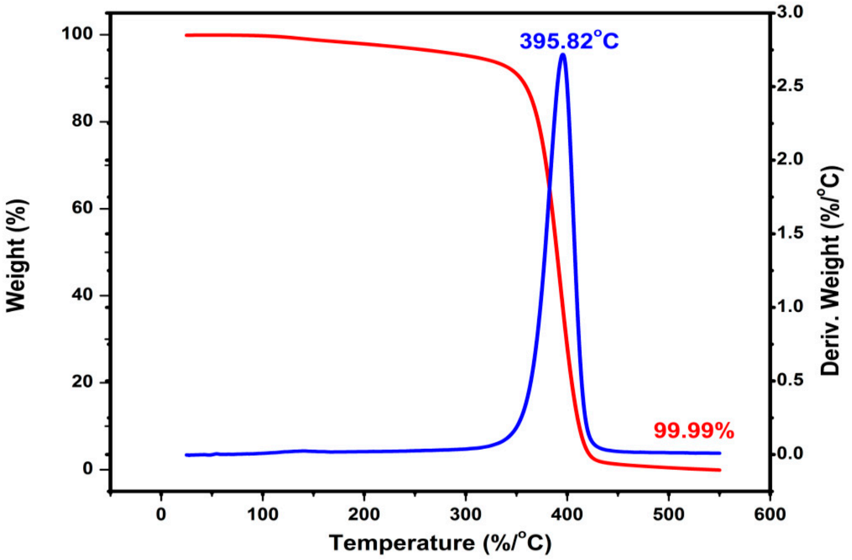

Figure S4. TGA/DTG curves of synthesized $PEGDA_{2000}$ monomer

Optimization results for the synthesis of nanogels

For the synthesized nanogels, the influence of the macromer composition (PEGDA2000, PEGDA700, and mixture of 75% PEGDA700 with 25% PEGDA2000) on the particle size distribution and polydispersity was followed for choosing the proper system to deliver particles ranging from 150-200 nm and with low polydispersity. DLS data are shown in Table S1. For the molecularly imprinted nanoparticles for which the enzyme was dissolved in water, the registered sizes were 152 – 163 nm while the ones with PLA2 dissolved in TRIS showed sizes in the range of 123 – 189 nm. The system with the most desired properties was MIP2-7 (T), or as noted in the main text MIP-LFNG (T), having also the lowest PDI in the series.

Table S1. *DLS results corresponding to the optimization study for the synthesis of MIP-LFNGs*

**Sample code**	**Average diameter of nanogels, (nm)**	**PDI**
NIP7	163 ± 7.7	0.326
NIP2	177 ± 0.5	0.444
NIP7-2 (NIP-LFNGs)	142 ± 1.1	0.326
MIP7(W)	152 ± 0.7	0.184
MIP2(W)	153 ± 0.5	0.183
MIP7-2(W) (MIP-LFNGs (W))	198 ± 3.91	0.375
MIP7(T)	123 ± 0.6	0.286
MIP2(T)	177 ± 0.7	0.227
MIP7-2(T) (MIP-LFNGs (T))	189 ± 9.2	**0.184**

The data collected from TEM for the nanogel systems with either $PEGDA_{700}$ or $PEGDA_{2000}$ alone, after purification and redispersion, recorded sizes between 140 – 500 nm

and even higher. Some of the gels were around 2 µm large and were agglomerated (see **Fig. S5**).

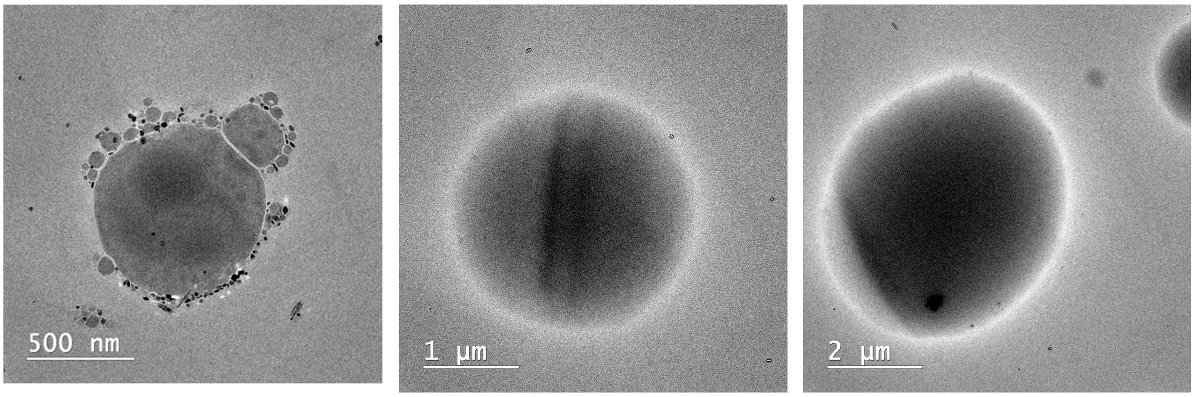

Figure S5. TEM images of redispersed NIP_7, MIP_7 (T) and MIP_7 (W)

Activity measurements of PLA2 and cytotoxicity

Table S1: PLA2 activity in solution and venom before and after contact with the LFNGs

		**C_f (U/mL) ± SD (*n*=2)**			
**Sample**		**15 min 490 nm**	**30 min 490 nm**	**15 min 450 nm**	**30 min 450 nm**
**PLA2 solution**	**NIP-LFNG**	17.9±2.9	8.7±12.3	21.0±13.4	10.4±14.7
	**MIP-LFNG (W)**	11.9±8.7	4.3±6.1	15.0±8.1	7.4±10.5
	**MIP-LFNG (T)**	328.8±5.7	353.6±7.9	319.6±4	348.8±6.8
**Venom**	**NIP-LFNG**	424.8±19.2	458.0±34.5	423.2±22.6	448.4±30.0
	**MIP-LFNG (W)**	400.0±11.3	432.0±5.7	393.2±15.3	422.0±8.5
	**MIP-LFNG (T)**	420.4±8.5	470.0±5.1	413.2±11.9	452.8±4.5
		**C_i (U/mL) ± SD (*n*=2)**			
**PLA2 solution***		378.4±9.1	403.6±19.8	370.8±7.4	394.0±16.4

**Venom**[**]	436.0±3.4	471.6±4.0	436.4±4.0	457.6±4.5

[*] *C_i (U/mL) is the initial concentration (activity of PLA2 in 0.1 mg/mL PLA2 feed solution)*

[**] *C_i (U/mL) is the initial concentration (activity of PLA2 in 1 mg/mL bee venom feed solution)*

Table S2: Cell Viability of L929 cell line after 24 H exposure to various dilutions of LFNGs

**Sample code** / **Dilution**	**Cell Viability (%) ± SD (*n*=3)**[*]		
	**MIP-LFNG (W)**	**MIP-LFNG (T)**	**NIP**
**D 1/4**	100.8±1.3	98.0±1.3	109.0±4.4
**D 1/8**	99.9±0.2	102.0±4.5	108.0±0.5
**D 1/16**	97.6±1.7	100.0±3.3	105±2.8
**D 1/32**	99.3±1.3	101.0±2.8	107±0.2

[*]The mean value of 5 measurements for the reference cells was 100.0±2.0
